# Caspofungin as Salvage Therapy for Pneumocystis Pneumonia in a Heart Transplant Recipient

**Published:** 2018-03

**Authors:** Sara Abolghasemi, Babak Sharif-Kashani, Farah Naghashzadeh, Majid Marjani, Afshin Moniri, Atousa Doroudinia, Payam Tabarsi

**Affiliations:** 1 Clinical Tuberculosis and Epidemiology Research Center, National Research Institute of Tuberculosis and Lung Diseases (NRITLD), Shahid Beheshti University of Medical Sciences, Tehran, Iran; 2 Chronic Respiratory Disease Research Center, NRITLD, Shahid Beheshti University of Medical Sciences, Tehran, Iran

**Keywords:** Pneumocystis Pneumonia, Chronic renal failure, Caspofungin

## Abstract

Pneumocystis pneumonia (PCP) is a common opportunistic infection in immunocompromised patients. In general, clinical response to therapy with cotrimoxazole is excellent. However, therapy may be limited by side effects or treatment failure. We present a case of PCP in a 35-year-old male patient with history of heart transplantation and renal failure who was admitted with a 10-day history of fever, nonproductive cough and elevated level of creatinine with a diagnosis of PCP confirmed by chest radiography and in bronchoalveolar lavage specimens. He was treated with trimethoprim-sulphamethoxazole (SMZ/TMP) and primaquine but treatment was completed with reduced dosage of cotrimoxazole, primaquine and with the addition of caspofungin. This therapy was effective and without any adverse effects in a patient with elevated level of creatinine.

## INTRODUCTION

Pneumocystis pneumonia (PCP) is one of the most current opportunistic, critical and life-threatening infection in immune compromised patient (1,2), Such as patients with connective tissue diseases, AIDS, history of transplantation and hematological malignancies (3).

The typical radiographic finding among patients who are infected with PCP is bilateral perihilar interstitial infiltration (4). The less common radiologic findings contain multiple or solitary nodules, upper-lobe infiltration, pneumatoceles and pneumothorax. Also pleural effusion and thoracic lymphadenopathy are seen rarely (5).

The first-line treatment for PCP infection is cotrimoxazole with various adverse effects, including leukopenia, rash, interstitial nephritis and thrombocytopenia (6). So we cannot prescribe it for patients with renal failure and creatinine level rising.

Caspofungin is a beta-1, 3-glucan synthesis inhibitor (antifungal) which in experimental animals has shown activity against PCP (7), but the clinical trials about this matter are rare (8–12). We here report our clinical experience with caspofungin in salvage treatment of PCP in a young man with a history of heart trantplantation and CRF; who referred owing to 10-day history of fever and nonproductive cough. We present our experience of the caspofungin consumption in the context of PCP in immune compromised individual with creatinine level rising and dramatic response.

## CASE SUMMARIES

A 35-year-old man, who had received heart transplant 3.5 years previously, was admitted to our hospital with 10-day history of fever and nonproductive cough. He had no history of myalgia, upper respiratory, gastrointestinal symptoms, headache and dyspnea. He had received empiric ciprofloxacin as an outpatient without improvement. His past medical history included heart transplantation 3.5 years ago due to dilated cardiomyopathy. He had chronic renal failure (CRF) following transplantation but his serum creatinine had risen from a baseline of 2.0 mg/dl to 5–6 over the past 8 months. His immunosuppression included tacrolimus 0.5mg at morning and 1 mg at night with a level of 5–6ng/ml, mycophenolate mofetil 500 mg twice daily and prednisolone 7.5 mg once daily, He had received valgancyclovir and itraconasole for CMV and fungal prophylaxis for 3months after transplantation and cortrimoxazole(trimethoprim-sulfametoxazole) until 6 months before presentation; cotrimoxazole was discontinued due to renal failure.

On examination his respiratory rate was 20 breaths per minute and his oxygen saturations were 82% without oxygen therapy. His temperature was 39 °C and blood pressure was 120/80 mmHg. Respiratory auscultation was normal and other physical examinations were unremarkable.

Arterial blood gas showed hypoxemia and blood count was normal without leukocytosis, Cr and BUN was 7.7 and 85 mg/dL respectively, LDH was 704 IU/L, other biochemistry and electrolytes were unremarkable, Respiratory secretions were tested negative for Influenza and other respiratory viruses. He had negative CMV serum PCR. Echocardiography was normal.

**Figure 1.**
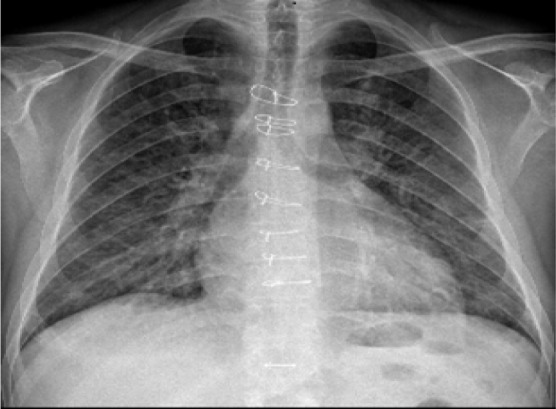
Chest radiography of patient

The results of chest radiography on admission and a subsequent computed tomography scan revealed bilateral diffuse infiltrates. (Figure 1)

Ceftriaxone, Azithromycin and oseltamivir had been initiated at admission and clindamycin with primaquine and intravascular hydrocortisone was added when Pneumocystis pneumonia (PCP) infection was suggested by the chest CT scan. Bronchoalveolar lavage (BAL) was performed. BAL was positive for *Klebsiella pneumonia*, PCR for CMV was positive but negative for HSV and EBV PCP monoclonal antibody, mycology smear and culture and galactomannan assays were negative. Meropenem 500 mg TDS and valcyte 450 mg every other day was initiated. After 4 days the patient didn't improve and O_2_ saturation declined to 87% despite facemask O_2_. Therefore Cotrmoxazole at dosage of 800/160 mg TDS was added.

**Figure 2.**
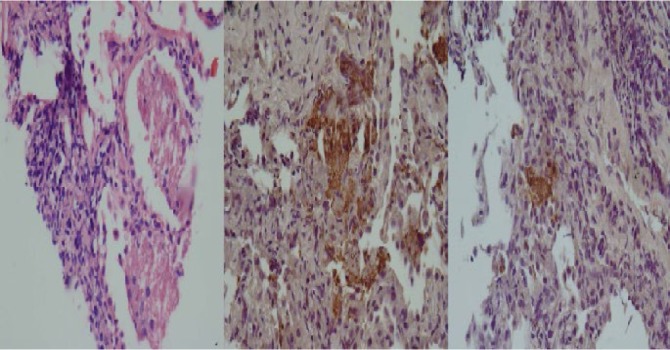
Histology of transbronchial biopsy of patient

Another broncoscopy with transbronchial lung biopsy (TBLB) was performed. Smear and culture of BAL was negative for acid fast bacilli and fungal organisms. At this time, bronchoalveolar lavage was negative for CMV PCR but positive for HSV1 PCR and histology showed many Pneumocyctis cysts in the lung biopsy (Figure 2) ten days after initiation of PCP-specific therapy. There was no evidence of CMV or other infections. The patient continued to deteriorate with O_2_ saturation on facemask decreased to 65%.

Caspofungin 70 mg in first day, then 50 mg daily was added and cotrimoxazole at dosage of 800/160 mg twice daily was continued, clindamycin, primaquine and meropenem were discontinued .

In second day after caspofungin administration patient's general condition became better and after 4 days o2 saturation increased. The patient completed 14-days of therapy with caspofungin and oral cotrimxazole. During treatment his Creatinine level decreased gradually to about 2.2 mg/dL. After 14 days of therapy the O_2_ saturation increased to 92% without any oxygen therapy.

Three weeks after completion of treatment, the patient was without any symptoms or signs. We continued cotrimoxazole at a dosage of 800/160 mg as a secondary prophylaxis.

## DISCUSSION

Pneumocystis pneumonia (PCP) is a leading cause of morbidity in immunocompromised individuals.

The diagnosis of PCP may be difficult without invasive diagnostic approaches including BAL or biopsy, notably in the non-AIDS immunocompromised host (13). In patients intolerant of cotrimoxazole, clindamycin plus primaquine, and atovaquone are second-line drugs and pentamidine intravenously is used in more severe form of disease (14).

Recent studies revealed that PCP is a fungal species with glucan-rich cyst wall (15). The effect of caspofungin against PCP has been shown in animal models (7), but there is limited clinical experience with this agent. This case may suggest that caspofungin in addition to reduced dose cotrimoxazole may provide advantages in patients intolerant of cotrimoxazole due to renal dysfunction (10,11)

Lobo and colleagues16 in their study reported that the administration of low doses of cotrimoxazole in combination with caspofungin provide an improved treatment protocol for PCP infection, in mice. Other studies also derived benefit from cotrimoxazole in combination with caspofungin in their patient (9,17)

Thus, caspofungin is recommended as a salvage treatment in patients with PCP (18).

Tu et al. (19) reported that the use of cotrimoxazole is associated with various side effects that may not be tolerated by critically ill patients so administration of caspofungin is recommended for PJP after solid organ transplantation. They showed that a combination of caspofungin and low-dose cotrimoxazole was useful in three cases of severe PCP in renal transplant recipients. In the current patient, combination therapy appears to be useful in the face of renal intolerance of full-dose cotrimoxazole.
